# Adult Hip Flexion Contracture due to Neurological Disease: A New Treatment Protocol—Surgical Treatment of Neurological Hip Flexion Contracture

**DOI:** 10.1155/2014/349014

**Published:** 2014-02-12

**Authors:** Alberto Nicodemo, Chiara Arrigoni, Andrea Bersano, Alessandro Massè

**Affiliations:** San Luigi Hospital, Department of Orthopedics and Traumatology, University of Turin Medical School, 10043 Orbassano, Italy

## Abstract

Congenital, traumatic, or extrinsic causes can lead people to paraplegia; some of these are potentially; reversible and others are not. Paraplegia can couse hip flexion contracture and, consequently, pressure sores, scoliosis, and hyperlordosis; lumbar and groin pain are strictly correlated. Scientific literature contains many studies about children hip flexion related to neurological diseases, mainly caused by cerebral palsy; only few papers focus on this complication in adults. In this study we report our experience on surgical treatment of adult hip flexion contracture due to neurological diseases; we have tried to outline an algorithm to choose the best treatment avoiding useless or too aggressive therapies. We present 5 cases of adult hips flexion due to neurological conditions treated following our algorithm. At 1-year-follow-up all patients had a good clinical outcome in terms of hip range of motion, pain and recovery of walking if possible. In conclusion we think that this algorithm could be a good guideline to treat these complex cases even if we need to treat more patients to confirm this theory. We believe also that postoperation physiotherapy it is useful in hip motility preservation, improvement of muscular function, and walking ability recovery when possible.

## 1. Introduction

Congenital, traumatic, or extrinsic causes can lead adults to paraplegia; some of these are potentially reversible as peripheral neuropathy drugs and alcohol induced [1] and others are not.

Depending on the level and on the extent of spinal damage, there can be a partial or complete loss of sensation and function in the affected limbs. Sexual impotence and urinary and fecal incontinence may also occur. Some of these patients can walk with aids, but most of them are dependent on wheelchair and forced to sitting position.

In these cases, as consequence of the prolonged sitting position, hips can develop a flexion attitude that results in dysplasia, dislocation, or ankylosis of the hips [2].

These patients may develop hip dislocation over time, with an incidence rate reported to be between 18 and 59% [3]. This is the consequence of muscular imbalance and capsular retraction that can cause a subluxation of the femoral head and finally a complete dislocation [4].

Fixed hip flexion can also lead to marked lumbar hyperlordosis, pelvic anteversion, and sometimes horizontal sacrum [5, 6]. This postural attitude can evoke lumbar and groin pain which can be associated with alterations of sensitivity. Moreover the decrease or loss of sensitivity combined with hip flexion and lumbar deviation causes sacral, gluteals and trochanteric pressure sores, skin infection, and sometimes even osteomyelitis.

Physiotherapy is very important to prevent these complications but when hip flexion is no more reversible, extension exercises can even worsen the lordosis due to the action of the iliopsoas. Even also surgical treatment aims to maintain sitting or walking ability, avoiding secondary skin ulcers and reducing lumbar hyperlordosis and pain. Many treatments have been proposed: tenotomies, myotomies, hip resection-arthroplasty, hip and acetabular osteotomies and total hip arthroplasty. Sometimes it is not easy to choose the right indication and in literature there are no clear evidences that can help surgeons' decision [7–10].

This study reports five cases of adult hip flexion due to neurological diseases treated in a single hospital according to an algorithm based both on literature and on personal experience of surgeons. The aim is to choose the best solution for each patient and to avoid useless or too aggressive surgery.

## 2. Clinical Cases

**Figure 1:**
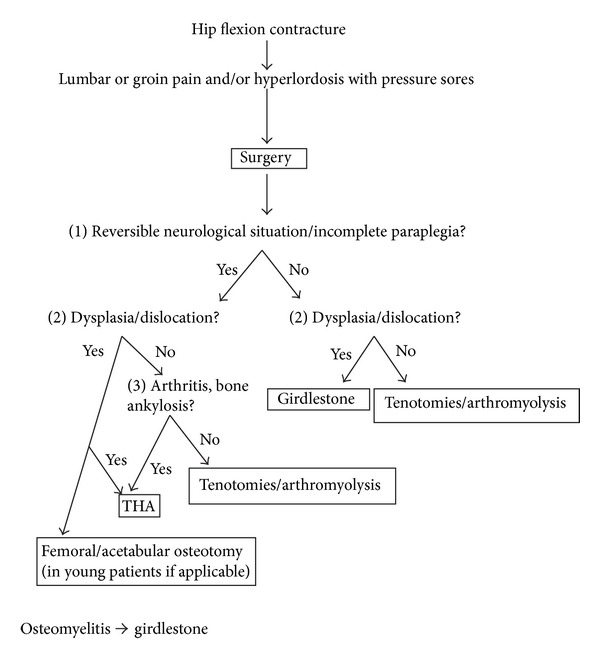
Our algorithm.

From 2007 to 2011 we treated in our institution five cases of hip flexion in adult patients with neurological disease. The choice of treatment has been carried out on the basis of physiological and pathological characteristics of patients (Table 1) and on a simplified algorithm specifically created to guide our therapeutic decisions in such cases (Figure 1).

We did not observe any major complication during or after surgery. After the operation, at least 3 months of physiotherapy was done to maintain the results (especially hip extension). In each case Celecoxib 200 mg was administered twice a day for 3 weeks as prophylaxis against heterotopic ossification.

The description of each case is given below.

### 2.1. Case 1

A 34-years-old man came to our attention with a diagnosis of spastic incomplete paraplegia due to a T12 spinal fracture reported in a car accident 3 years ago. He could not walk and was wheelchair-dependent since that moment. He complained of a severe lumbar and right groin pain and at physical examination he presented with a fixed right hip and knee flexion (resp., about 60° and 20°). X-rays and CT images revealed a posterior dislocation of the right femoral head, Brooker III ossifications about the right hip, and no evident abnormalities of the knee. We treated him by removing the ossifications, implanting a THA (total hip arthroplasty), and making a posterior capsulotomy of the right knee. At one-year-follow-up the patient presented with a good range of motion of the hip (passive flex-extension: 100°-0°-0°) and of the knee (passive flex-extension: 90°-0°-0°). Surprisingly he could walk with an atasso-spastic gait helped by 2 crutches for few meters; he had no skin sores and no more lumbar or groin pain.

### 2.2. Case 2

A 36-years-old woman presented with an incomplete paraplegia with a bilateral hip flexion contracture secondary to a severe spinal cord contusion reported in a car accident 2 years ago. She was wheelchair-dependent since that moment. She suffered from a severe groin and lumbar pain and at physical examination she had a hardly modifiable bilateral hip flexion (about 45°) due to a retraction of the anterior soft tissues. X-rays images did not show any bone or articular abnormalities. We treated her with a bilateral arthromyolysis through a Smith-Petersen access. After six months of intensive physiotherapy she returned to walk with one crutch; at one year follow up she had a normal range of motion of the hips, she could walk for 500 meters without crutch, she had no pressure sores and no more lumbar or groin pain.

### 2.3. Case 3

A 55-year-old man was referred to us for bilateral Brooker IV heterotopic ossifications caused by an alcoholic neuropathy. He complained of severe lumbar pain and limited groin pain. He had a bilateral hip ankylosis with a fixed flexion at about 50° and a fibrous ankylosis of right knee with 80° of flexion. He was moving in a wheelchair four years ago. The neurological examination showed that muscular contraction and limited active joint movement were possible. X-ray and CT-scan images showed signs of bilateral hip osteoarthritis and Brooker IV periarticular heterotopic ossifications. We treated him with a bilateral removal of the ossifications and THA through a posterior approach. An anterior soft tissues release of the right hip through a Smith-Petersen approach and posterior capsular release of the right knee were also necessary to obtain complete extension of hips and knees.

At one-year-follow-up he had a good range of motion of the hips with a residual bilateral flexion of 20°. The right knee had a residual flexion of 10°. He was able to walk for few meters with the help of two crutches. He had not pressure sores or no more lumbar, groin, or knee pain.

### 2.4. Case 4

A 28-years-old man had a complete paraplegia caused by a paraspinal Burkitt's lymphoma at the age of six. As a consequence he had an ankylosis in flexion of both hips.

At the age on ten he underwent multiple tenotomies around the left hip and six years later a resection of the right femoral head to treat an osteomyelitis secondary to pressure sores.

He complained lumbar pain not controlled by medication (daily intake of high dose of NSAID) and at physical examination he presented with a flexed left hip (more than 90°), marked lumbar hyperlordosis, and trochanteric pressure sores. X-ray images showed a posterior subluxation of left femoral head and a horizontal sacrum. Few months before a spine surgeon proposed to him a lumbosacral fusion to relieve his back pain, but he refused the operation. We treated him with an arthromyolisis and resection of the left femoral head through a Smith Petersen approach. After one year of follow up he had a residual hip flexion of 20°, pressure sores healed and the lumbar pain disappeared without the need of anymore operation.

### 2.5. Case 5

A 29-years-old man with a spastic quadriplegia with dystonia had an inveterate dislocation of the right hip. He underwent many tenotomies and four years before a spinal lumbosacral arthrodesis to treat the evolutionary scoliosis and hyperlordosis. He came to our attention because of the recurrence of severe lumbar pain. At physical examination he presented with fixed right hip flexion (about 90°) and sacral pressure sores. He moved in a wheelchair.

X-rays images showed a posterior and superior hip dislocation with neoacetabulum. We treated him with Girdlestone's operation through a posterior approach.

At one year of follow-up he had a residual hip flexion of 30° and a mild lumbar pain during prolonged sitting position. Pressure sores completely healed.

## 3. Discussion

Hip flexion contracture in adult paraplegic patient is a relatively rare condition and a combined strategy of precocious preventive physiotherapy and local botox injections and tenotomies can avoid aggressive treatment in most of the cases.

The key point of surgical treatment is the choice of the correct indication. Michaelis [10] had good results with iliopsoas and obliquus externus myotomy; Graham et al. [11] described 3 cases of recurrent dislocation of the hip in adult paraplegics successfully treated with open reduction and bone block augmentation of the acetabulum; Becker et al. [12] focused on periarticular ossification in paraplegics, reporting the results of six patients treated with a total hip replacement. He recommends avoiding femoral head resection in favour of total hip arthroplasty even in complete paraplegia, as the former has the risk of posterior trochanteric dislocation and consequent formation of pressure sores. But, on the other hand, in case of hip replacement, high dislocation rates, risk of infection (especially in presence of pressure sores), osteoporosis, loosening risk, and blood loss must be taken into account. All these studies have no statistically significant evidence due to the exiguity of the cases and to the difficulty having homogeneous examples.

Each of these cases slightly differs from the others in terms of clinical presentation, symptoms, and anatomo-pathological features. The lack of homogeneity in clinical cases imposes a careful evaluation of each case before treatment. Many factors have to be considered: age, neurological status, hips and knees articular assessment, spinal deformity, localization of pain, pressure sores, skin or deep infection, and other comorbidities. According to these features, we tried to choose the best treatment for each patient and we tried to resume them in a decisional algorithm that, at last, was successful in our experience in relieving lumbar and groin pain, improving skin condition, and restoring a good hip motility.

In three cases (cases 1, 2 and 3) patients were able to return to walk even if with limitations and aids. It is evident that their neurological condition allowed a residual possibility of active muscular contraction in the lower limbs.

**Figure 2:**
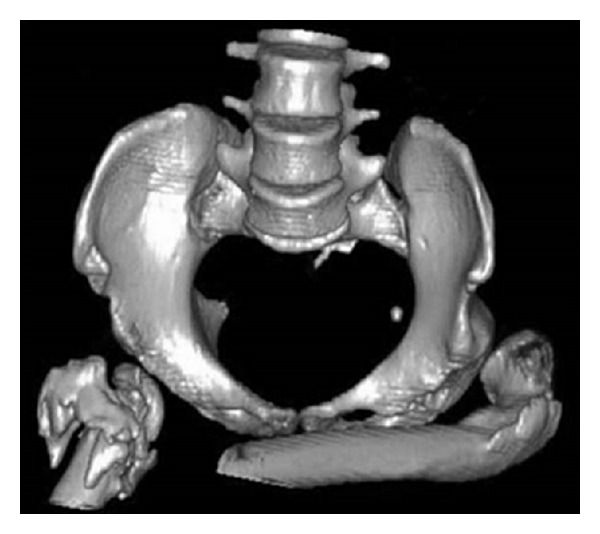
Case  4  preoperative CT.

**Figure 3:**
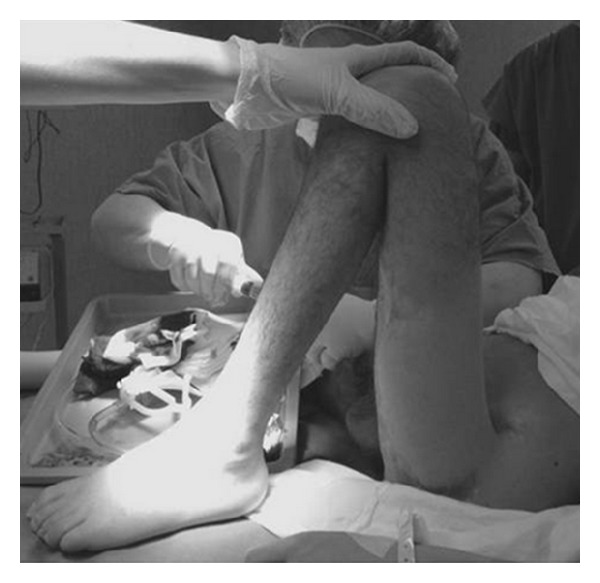
Case  4  Preoperative hip flexion contracture.

**Figure 4:**
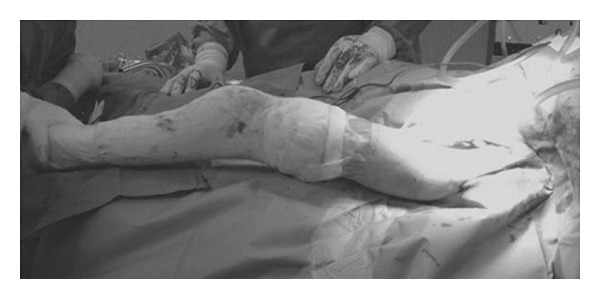
Case  4  Postoperative hip flexion.

In case 4, after resection-arthroplasty of the hip, back pain improved so that spinal surgery was no longer necessary even in presence of a horizontal sacrum (Figures 2, 3, and 4). On the other hand, in case 5, spinal arthrodesis did not resolve back pain, which was improved after resection-arthroplasty of the hip. We can say that lumbar pain should be assessed with much attention as it can be secondary to hip fixed flexion and probably in these cases spinal surgery should be considered only after hip surgical release.

All five patients had a good joint mobility at 1-year follow-up control (Table 1). Due to the insufficient sample we cannot draw statistically significant results.

## 4. Conclusion

There are few studies about the treatment of hip flexion in paraplegic adult patients. Therefore there is no a clear protocol to be used in these cases.

Most of the authors cited above propose only a single surgical procedure but we think that it is not a correct approach related to the variability of the pathological presentations.

We tried to make a decisional algorithm based both on our experience and on the few indications found in scientific literature. We decided to start our algorithm from the clinical evaluation of the patient, taking into special account the residual possibility of walking ability. Some patients generically defined as paraplegic, at a deeper assessment, show a residual active muscular contraction due to a partial neurological recovery or to the reversibility of their condition (i.e., alcoholic neuropathy). If some active muscular activity is possible, the joint must be preserved, when possible, or replaced, when is not. On the other hand, when paraplegia is complete and irreversible, a femoral head resection can be considered; even if it looks like more demolitive, it has fewer potential complications than arthroplasty (i.e., infection, dislocation, mobilization, etc).

As happens for the surgery, only specialized personnel should carry out preoperative and postoperative physiotherapy. The perfect knowledge of hip anatomy and neurological situation of each patient is the guide to obtain and maintain good results and to avoid harmful therapies, for example, passive hyperextension in flexed ankylosis, which cannot improve hip motion, but it could worsen hyper-lordosis causing back pain.

The application of this algorithm gave good results in all our cases and probably could help surgeons to choose the best treatment in terms of cost-effectiveness. We know that our follow-up is oversimplified compared to the heterogeneity of the clinical manifestation pre-and postoperation so it is clear that we would need to treat more patients to validate its utility. We believe also that physiotherapy has a significant role in the preservation of postoperative hip motility, improvement of muscular function, and walking ability recovery when possible.

## Figures and Tables

**Table 1:** Patients and outcome.

Case	Age	Gender	Diagnosis	Flexed hip	Joint	Treatment	PFF	PRF	PWA Yes/No
1	34	M	Incomplete paraplegia	Right	HO + post dislocation	THA	60°	0°	Y
2	36	F	Incomplete paraplegia	Bilateral	Normal	Anterior AM	45°	0°	Y
3	55	M	Alcoholic neuropaty	Bilateral	HO + OA	THA + Anterior AM Right hip	45°	20°	Y
4	28	M	Incomplete paraplegia	Left	Dysplasia	Anterior AM + Girdlestone	80°	20°	N
5	29	M	Spastic quadriplegia with dystonia	Right	Post dislocation	Posterior AM + Girdlestone	90°	30°	N

PFF: preop hip fixed flexion.

PRF: postop hip residual flexion.

PWA: postop walking ability.

AM: arthromyolysis.

HO: heterotopic ossifications.

OA: osteoarthritis.
